# Analysis of Iranian health workforce emigration based on a system dynamics approach: a study protocol

**DOI:** 10.1080/16549716.2024.2370095

**Published:** 2024-07-12

**Authors:** Hamideh Keyvani, Reza Majdzadeh, Esmaeil Khedmati Morasae, Leila Doshmangir

**Affiliations:** a Department of Health Policy & Management, School of Management and Medical Informatics, Tabriz University of Medical Sciences, Tabriz, Iran; b School of Health and Social Care, University of Essex Colchester, Colchester, UK; c Complex Systems Science, Research Fellow in Complex Systems and Policy, Faculty of Environment, Science, and Economy, University of Exeter, Exeter, UK; d Department of Health Policy & Management, Tabriz Health Services Management Research Center, School of Management and Medical Informatics, Tabriz University of Medical Sciences, Tabriz, Iran

**Keywords:** Emigration, health worker, health human resources, system dynamics, policy analysis

## Abstract

**Background:**

Emigration of health workers has emerged as a significant obstacle in Iran, compelling policymakers to implement a diverse range of interventions and reforms to enhance healthcare services. It is imperative to comprehend the efficacy of emigration control interventions. To explore the intricate dynamics of elite emigration, this study employs a system dynamics modeling approach. The objective is to scrutinize Iranian health workers’ emigration, examine the relationships, and evaluate the impact of various factors involved.

**Objectives:**

The general purpose of this study is to analyze the policy interventions affecting the emigration of human resources in the field of health in Iran based on the system’s dynamic approach.

**Method:**

The research consists of four phases including analyzing the emigration status of health workers in developing countries, studying health personnel emigration in Iran, developing a System Dynamics (SD) model, and determining evidence-based policy interventions to address health worker emigration. These phases involve realist review, document analysis, qualitative interviews, data integration, and policy scenario planning. The primary objective is to gain a profound understanding of the underlying causes, mechanisms, and consequences of migration, as well as assess the impact of policies, in order to prioritize effective interventions.

**Results:**

It seems that the SD model developed in this study can highlight the interconnectedness of various factors that influence health worker emigration, including demographic changes, economic conditions, and characteristics of healthcare systems.

**Conclusion:**

This study uses a systems dynamics approach to analyze health worker emigration from Iran, focusing on policies that promote retention and explore the implications of emigration on the healthcare system. By examining interrelationships and feedback loops within the healthcare system and socioeconomic factors, the study aims to identify effective policy interventions that can mitigate the negative effects of emigration.

## Paper context

**Main findings:** The study reveals a complex web of factors driving the emigration of Iranian health workers, and along with the Investigating the effectiveness of different interventions, the aim of this research is to develop sustainable solutions to deal with the emigration of health workers.

**Added knowledge:** This research adds to the existing literature by employing a system dynamics approach to model the multifaceted nature of health workforce emigration in Iran, offering a novel perspective on policy intervention analysis.

**Global health impact for policy and action:** The findings underscore the necessity for comprehensive policy strategies that address the root causes of health worker emigration and can inform global efforts to retain healthcare professionals and ensure sustainable healthcare systems.

## Background

The availability of specialized human resources is crucial for healthcare systems, impacting their sustainability and effectiveness [1]. In the healthcare domain, the adequacy and suitability of specialized human resources present critical considerations, encompassing factors such as their numbers, classifications, allocation methods, and effectiveness [2].

The migration of healthcare professionals, commonly known as the medical brain drain, poses a global labor crisis in the healthcare industry [3]. This phenomenon specifically refers to the large-scale emigration of skilled and capable healthcare workers, including physicians, nurses, and midwives, from low-income countries to high-income nations [4]. The global health workforce is already unevenly distributed, and this situation worsens due to the significant outflow of doctors and nurses, driven by poor working and living conditions and aggressive recruitment efforts in affluent countries [5].

While the reasons for healthcare worker migration vary across nations, some common motives include a lack of basic amenities, high unemployment rates, inadequate housing, low pay, uncertain career prospects, workplace hostility, political instability, and armed conflict – all considered pressure factors contributing to healthcare worker migration [6]. Conversely, the availability of suitable amenities, low unemployment rates, higher wages, positive career prospects, improved working conditions, and job satisfaction serve as key incentives that can offset the lack of resources in the healthcare worker’s country of origin [7,8]. The worldwide trend of skilled healthcare professionals migrating has surged in recent years, resulting in a notable increase of over 60% in the count of immigrant physicians and nurses employed in OECD nations from 2010 to 2020 [9]. The proportion of foreign skilled human resources in the healthcare systems of the United Kingdom, the United States, and the member states of the European Union stands at 12%, 17%, and 11%, respectively [10].

Various nations have explored different approaches to manage the migration of healthcare professionals. For instance, Zambia has implemented policies to retain health workers by offering financial incentives, including retention allowances for university graduates, uniform maintenance allowances for nurses, and housing allowances [11]. In Bangladesh, health sector reforms, such as ensuring reliable and prompt payment of salaries, have had positive motivational effects [12]. In Cairo, strategies have been proposed for affluent nations to increase their own production of healthcare workers, reducing reliance on importing them from low- and middle-income nations, and creating methods to compensate these nations in times of healthcare worker loss [13].

The emigration of healthcare professionals from Iran has become a significant obstacle in recent times. The International Monetary Fund has disclosed that approximately 180,000 knowledgeable Iranians leave the nation annually in pursuit of more favorable livelihood prospects, resulting in an annual outflow of 50 billion dollars of foreign currency from the country [14]. In 2022, the Iran Migration Observatory conducted a survey which revealed that 54% of doctors and nurses expressed a strong desire to leave the country. Of those surveyed, 24% had already decided to emigrate and 7.5% had taken concrete steps toward doing so. The survey indicates a high level of intention to emigrate among healthcare professionals in Iran [15].

The migration of healthcare workers in Iran is a significant concern that has been extensively studied in numerous scholarly articles. Various factors influence the inclination to migrate among skilled healthcare professionals in Iran, including age, work experience, employment status, marital status, proficiency in a foreign language, and prior experience of residing abroad [16]. Asadi et al. [17] identified five primary determinants that impact the migration of healthcare professionals in Iran’s healthcare system: structural, occupational, personal, socio-political, and economic factors. In Hajiani’s study [18], the motivations behind the migration of Iranian dentists to Canada were classified into four categories including socio-political, economic, professional, and personal factors. Factors influencing the inclination to migrate among medical students at Tehran University of Medical Sciences, Tehran, Iran include gender, pre-university study region, parents’ educational attainment, having relatives abroad, experience of traveling to foreign countries, foreign language skills, number of published articles, and membership in Iran’s National Elite Foundation [19]. Another study found that the intention to migrate among nurses in Iran had a weak correlation with workload and an inverse correlation with a healthy work environment [20]. Sanctions have contributed to the healthcare crisis in Iran by restricting access to medicines and medical devices and impacting the overall healthcare delivery [21]. In addition, organizational inefficiencies within Iranian hospitals, such as budgeting and payment systems, and a lack of managerial skills, have also been identified as contributing factors [22]. Various studies have shown that health workforce migration in Iran is a complex issue influenced by both internal factors, such as socio-political dissatisfaction, economic conditions, and the structure of the healthcare system, and external factors, such as global market dynamics and opportunities abroad [23,24]. Mitigating healthcare professional migration from Iran requires the implementation of targeted policies and international cooperation to address the underlying factors.

Various studies have comprehensively addressed the reasons and influencing factors regarding health workforce migration from Iran. However, this study focuses on examining the interventions implemented to tackle this challenge. Previous research has paid less attention to this topic.

Additionally, this study investigates the experiences of other countries, reviews their approaches and policies in attracting and retaining health workforce. Drawing lessons from the experiences of other countries and comprehending the effectiveness of diverse interventions can offer guidance to countries encountering similar challenges in health workforce migration. Policymakers and health system planners can develop more efficient strategies to address health workforce migration and enhance the quality of healthcare services in their respective nations. Efforts to improve healthcare human resources recruitment and retention in Iran remain insufficient. Elite migration is a complex and evolving phenomenon influenced by various factors. To understand the relationships and impacts of these factors, a system dynamic modeling approach can be utilized. This methodology involves constructing a mathematical model to analyze the system’s behavior over time. By employing this approach, the aim is to gain a deeper understanding of the dynamic mechanisms underlying observed phenomena, identify root causes, and predict future trends based on current conditions. This comprehensive approach addresses the fundamental causes of the issue, rather than solely treating its surface-level consequences [25].

Evidence-based policymaking is crucial for effective health system reforms. Stakeholders in the health system, including policymakers, managers, and employees, aim to enhance the quality, safety, effectiveness, and efficiency of healthcare services by adopting and implementing interventions and reforms from other countries. Contextual factors significantly impact the outcomes and progress of these initiatives. Hence, it is essential to recognize the rationale and methodology guiding healthcare interventions [26]. This modus operandi operates by establishing causal correlations between intervention programs, context, mechanism, and outcome (PCMO). Its aim is to provide evidence on identifying optimal interventions and suitable implementation methodologies in accordance with the circumstances of each locale [27].

This study investigates healthcare human resources migration in Iran using a system dynamics model. It starts with a realist review to examine reasons for health worker emigration and interventions in low- and middle-income countries. By analyzing evidence and data, the study aims to understand the rationale behind successful or unsuccessful policy implementation. Connections between variables will be established through systems dynamics modeling, leading to recommendations for more suitable approaches in Iran’s healthcare system.

## Methods

### Aim and objectives

This investigation aims to analyze the emigration of Iranian healthcare workforce using a systems dynamics approach, with four key objectives:

Realist Review: Conducting a comprehensive literature review to understand health worker emigration in developing countries, including reasons, context, mechanisms, and policies.

Qualitative Interviews and Documents Review: Gathering insights specific to Iran through qualitative interviews and document systematic review.

System Dynamics (SD) Model Development: Building an SD model based on the findings from the previous phases, including scenario analysis.

Evidence-Based Policy Interventions: Identifying and prioritizing evidence-based policy interventions to effectively tackle health worker migration.

The proposed approach is a multi-method approach guided by the principles of methodological pluralism. This allows for the combination of different research methods to comprehensively address complex research questions. In this study, multiple methods are used to triangulate the data and provide a comprehensive understanding of health workforce migration in Iran, including its causes and the interventions implemented to address it.

### Study design

#### Phase 1: realist evaluation

A realist evaluation survey will examine health worker migration and policy interventions in low- and middle-income countries. The study will follow Pawson et al.‘s six-step approach, including developing an initial program theory, searching for evidence, selecting and appraising documents, extracting data, synthesizing evidence, and presenting a revised program theory. These countries have diverse economic, social, and cultural structures, along with varying laws and guidelines. See Table 1 for a detailed outline of the process [28].

**Table 1.** Methodological steps to complete the realist review.

Steps	Task(s)
1. Clarifying the initial program theory	Search for initial theories and then consult with experts
2. Search strategy	Search electronic databases using keywords and Medical Subject Heading (MeSH) terms
3. Select and appraise documents	● Use inclusion and exclusion criteria to screen for relevant abstracts, articles, and reports ● Retrieve full-text of articles and reports
4. Extract data	● Use standardized tool to extract relevant data ● Search reference lists by hand for additional potentially relevant articles and reports
5. Analysis and synthesis process	● Analyze data for content and outcome patterns and synthesize mechanisms ● NB: Realist reviews follow an iterative search process, so revise Step 2 (i.e., search strategy) if relevant
6. Present and disseminate revised program theory	Present and refine revised theoretical findings with relevant stakeholders and experts

##### Phase 1, step 1— developing an initial program theory

The review aims to elucidate one or more fundamental theories underlying the intervention program. The basic theory will be explained using the concepts of Plan, Context, Mechanism, and Outcome. Relevant theories will be identified through an exploratory and inductive review of articles, reports, official documents on migration policies, and insights from key actors. A framework based on the context-mechanism-outcome principle will be developed and discussed with professors. The research will focus on contextual factors influencing program implementation and examine the interrelationship between mechanisms, outcomes, and contextual dimensions. Comparative analysis between studies will be used to achieve this objective

##### Phase 1, step 2— searching for evidence

The next step involves systematically searching for primary studies that are relevant to the program theory established in Step 1. Databases like PubMed, Scopus, MEDLINE, Science Direct, WHO Global Health Observatory, Google Scholar search engine and Human Resources for Health Journal will be searched using a devised search strategy. The search will include indexing terms related to health worker migration, program theory mechanisms, and specific geographic focus (e.g. low- and middle-income nations as classified by the World Bank) [29]. English key terms and their variations will be used, and retrieved documents will be assessed based on inclusion/exclusion criteria. (see Tables 2 and 3). The search strategy will be refined based on initial results, and a snowball sampling approach will be used to explore references of retained articles [30]. Unpublished and grey literature will also be considered. Reference lists will be screened for additional relevant literature until saturation. Mendeley will be used for reference management.

**Table 2.** List of key words for the document search strategy.

OR	OR	OR	OR	Themes and expressions (grey literature)
AND				
Health worker, Health human resources, Human resource Staff	management, policies, intervention, regulation, Health policy	Developing countr*, Low income countr*, LMIC*, China, India, Brazil, Russia, African countries, Asian countries	Shortage, retention, motivation	Migration, Emigration, Brain drain, National health programs

**Table 3.** Inclusion and exclusion criteria.

Inclusion criteria	Exclusion criteria
● Main focus on emigration ● Represents the entire collection ● Laws and official reports of the relevant country ● Intervention is to control health worker migration ● National migration policy addressed in the document	● Address a different issue than health worker migration ● Lack of valid sources ● Published in languages other than English ● Inaccessible complete texts ● Focus on emigration control by non-governmental organizations

##### Phase 1, step 3— selecting and appraising documents

Selecting and Appraising Documents: Unlike conventional systematic reviews, realist reviews do not require the evaluation of study designs based on an evidence hierarchy. Instead, quality assessment is conducted from a heuristic perspective to enhance C-M-O configurations and must answer the question: ‘Does this study offer adequate evidence to contribute to the synthesis?’ Each study will be evaluated based on its ability to clarify configurations, rather than as a singular unit of analysis [31]. To ensure the review’s credibility and trustworthiness, two independent reviewers will evaluate the rigor and relevance of each study’s potential contribution to the development of the program theory. Rigor and relevance will be assessed by scrutinizing if the study inferences are grounded in evidence or the author’s opinion. Any discrepancies during the screening process will be resolved by a third reviewer if necessary.

##### Phase 1, step 4— extracting data

Data extraction will involve capturing relevant study information in a Microsoft Excel spreadsheet. This includes details like author’s name, publication year, study setting, implementing organization, and study type. The extraction process will be guided by specific domains based on the initial program theory. These domains will cover explanatory accounts in CMO configuration, supporting or contradicting aspects of the program theory, and notes on mechanisms, context, outcomes, and program design elements. The domains will be continuously reviewed as the study progresses, allowing for the identification of new effects not accounted for in the original theory and the formulation of more focused research questions.

##### Phase 1, step 5— analysis and synthesis of data

In this study, each individual research will be carefully examined to determine if the emerging findings support, oppose, or reinterpret the initial program theory. The evidence will be thoroughly analyzed to understand the complex connections between context, mechanisms, and outcomes, both intended and unintended. Recurrent relationships among context, mechanisms, and outcomes will be identified, along with how similar mechanisms operate in different contexts. The review will also assess any contradictory examples and the strengths and weaknesses of the research methods employed. Ultimately, the findings will refine the theory, shedding light on the connections between contextual factors, mechanisms, and outcomes of migration interventions.

##### Phase 1, step 6— generating a revised program theory

The revised program theory will be explained using textual and diagrammatical means, and its evaluation will be reported following the established guidelines of the Realist and Meta-narrative Evidence Synthesis (RAMESES) consortium [32]. The involvement of stakeholders is imperative to verify emergent findings and facilitate dissemination activities. The identification and analysis of emerging findings and context-mechanism-outcome (CMO) configurations will be presented and discussed during conference sessions. Ultimately, the final presentation of the theory will be determined as a result of these stakeholder discussions.

#### Phase 2: context analysis

In this phase, the analysis of the trend and interventions to control the migration of health workers in Iran is carried out in two steps, which are explained in detail below.

##### Phase 2, step 1 – reviewing documentation

Asystematic review examines health workforce migration in Iran through document analysis. Multiple search engines and databases are used, including Google, Google Scholar, scientific databases, and data from organizations like the World Health Organization and the Ministry of Health. The review follows specific inclusion and exclusion criteria for policy documents, focusing on valid, citable, and relevant policies related to health workforce migration in Iran. The analysis employs content or thematic analysis to identify patterns and themes in the data. Common themes and trends across policies are identified and interpreted within the research question’s context. Strengths, limitations, and implications for practice, policy, and future research are considered in the findings.

##### Phase 2, step 2 – conducting interviews

Interviews: In this phase, a qualitative research design will be used, conducting semi-structured interviews with health professionals who have experienced migration. Participants will be recruited through purpose-based sampling and snowball approach until data saturation is reached. Interviews will be conducted in person or online, focusing on motivating factors for migration, challenges encountered, and the acculturation process. Participants’ opinions on policies and solutions to improve the retention of the health workforce will also be explored. Interviews will be audio-recorded and transcribed, and content analysis will be used to analyze the qualitative data. Additionally, qualitative interviews with managers and decision-makers in the health field, particularly those who are key and influential in addressing health personnel migration, will be conducted. Topic analysis will be performed to identify common topics, categories, patterns, and relationships between categories, ultimately leading to the determination of solutions by combining and interpreting the findings.

#### Phase 3: modelling using system dynamics approach

**Figure 1.**
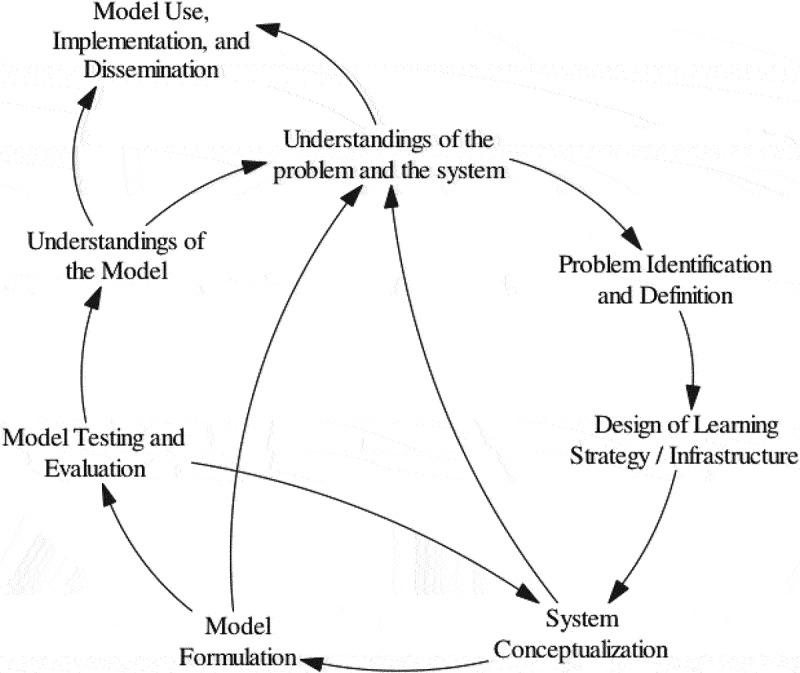
Overview of a system dynamics modeling approach.

The impact of policies on human resources migration is investigated through a system dynamics modeling approach. A conceptual model is formulated based on causal relationships between primary and secondary factors, which are revised and validated by experts. Primary factors like age, gender, and ethnicity, as well as secondary dimensions like income and social situation, are examined to understand their influence on health worker migration [33]. Experts from the healthcare field, including experienced immigrants and managers overseeing migration, contribute to the model development. Using the conceptual model, a dynamic model of the emigration process and its determinants would be construct. This model serves as a platform to explore the effects of policies on healthcare workforce migration and the healthcare system in Iran. Vensim software will utilize for modeling. The process involves six key activities, as depicted in Figure 1 [34]. These activities are as follows: (1) problem identification and definition, (2) system conceptualization, (3) model formulation, (4) model testing and evaluation, (5) model use, implementation, and dissemination, and (6) design of learning strategy/infrastructure [34].

##### Phase 3, step 1 – problem identification and definition

This phase involves conceptualizing the problem and defining the model’s boundaries. The focus of this system dynamics model is the emigration of the Iranian health workforce and its impact on the healthcare system. The model specifically investigates interventions affecting the emigration of doctors, nurses, and university faculty members, informed by document reviews, realist reviews, and interviews with individuals familiar with the emigration process. Policies are essential in understanding their potential impact on factors influencing emigration, guiding the model’s scope and determining what aspects and factors are included or excluded. Developing a system dynamics model of health workforce migration enables policymakers and healthcare stakeholders to comprehend the complex dynamics involved and identify potential interventions to mitigate its effects.

##### Phase 3, step 2 – system conceptualization

**Figure 2.**
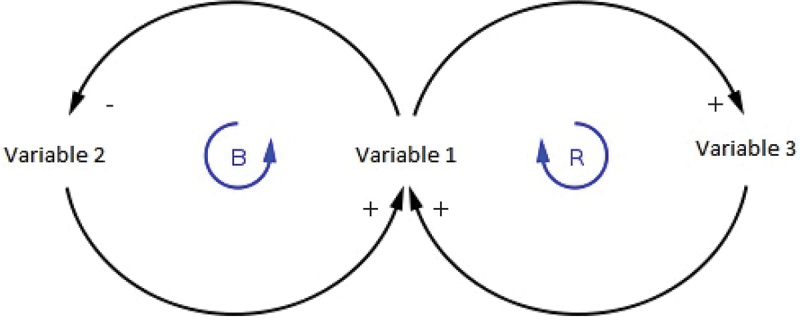
A hypothetical causal loop diagram used to demonstrate the dynamics of a system components.

In this step, the system conceptualization is enhanced by identifying the key components and relationships that were discussed and uncovered in the first step. It involves developing a causal loop diagram (CLD) that illustrates the causal relationships and feedback loops between the different components of the system (Figure 2). The diagramming process will be informed by study participants and may undergo repeated revision until the diagram is considered acceptable and closely represents the real phenomenon by the experts/participants. The causal loop diagraming is a process of constructing and diagramming a set of interlocking/interrelating dynamic hypotheses regarding the mechanisms of the emigration phenomenon. The CLD illustrates how emigration factors or determinants influence each other and how such influences reverberate across the system, leading to potential systemic repercussions.

##### Phase 3, step 3– model formulation

**Figure 3.**
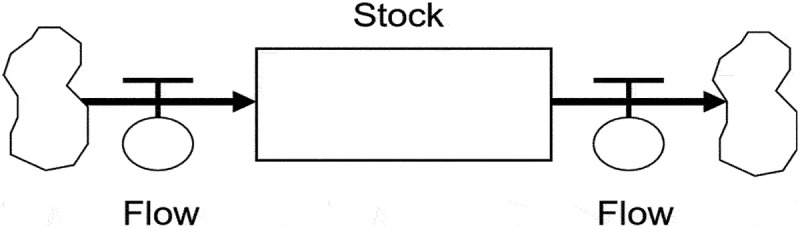
Minimal stock-and-flow diagram (SFD).

**Figure 4.**
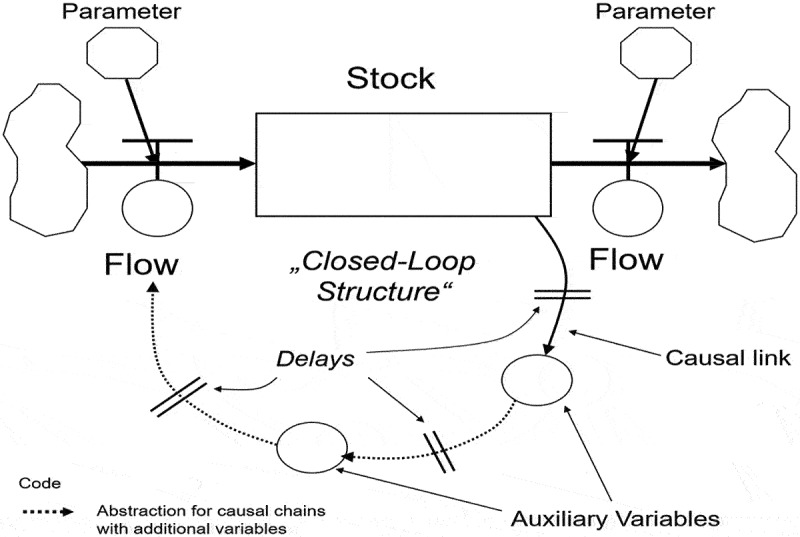
Closed-loop stock-and-flow diagram (SFD).

In this step, the conceptual model (CLD) is translated into a mathematical model suitable for simulating the problem. The mathematical model comprises equations that describe the system’s behavior over time. Stocks, flows, model parameters, and auxiliary variables are the fundamental components of the model formulation (Figures 3 and 4) [35]. Stocks in a system dynamics model represent accumulated variables or factors, providing a snapshot of the system’s status at specific time points. For example, in our study, a stock could represent the number of emigrated health workers over the past decade. Flows, on the other hand, represent the rates at which stocks change, indicating the variables that cause stocks to increase or decrease over time. In our study, a flow might represent the rate of health worker emigration per day/month/year. Feedback relationships between stocks and flows exist, where changes in stocks can influence and alter the flows that contribute to the stocks. Parameters are fixed values in the model that influence system behavior, typically determined using available data, expert knowledge, or other sources. Auxiliary variables are intermediate factors resulting from stocks or flows, linking them to other elements. These model components are derived from the pre-constructed causal loop diagram (CLD), and mathematical equations, such as integral and differential equations, define the relationships between stocks, flows, parameters, and auxiliary variables.

##### Phase 3, Step 4 – model testing and evaluation

In this step, the model is tested and evaluated after incorporating data (historical and estimated) into it, ensuring its accuracy in representing the problem being modeled. This step involves variable calibration and model validation. Calibration includes determining the numerical values of the variables or factors in the model. Numerical data can be obtained from available data resources (historical data) or estimated for missing data. If needed, data estimation involves using expert knowledge and specific statistical methods (e.g. Bayesian statistics) to estimate the probability distribution of missing values over time.

There are two ways to test and validate an SD model: structural and behavioral validation. Structural validation involves reviewing the model with stakeholders/experts to ensure the model structure accurately captures the main aspects of the real-world problem. Behavioral validation entails using statistical tests (e.g. mean differences) to compare the model outputs with real trends (e.g. whether the model output on the trend of emigration of Iranian healthcare staff over the last decade aligns closely with the real trend). If there are significant discrepancies between model outputs and real trends, the modelers adjust the model, in consultation with experts and stakeholders, until it replicates the real trend.

Sensitivity analysis is also conducted to assess and understand the impact of changes in parameters, inputs, and assumptions on the output and behavior of the system. The primary goal of sensitivity analysis is to determine whether the model behaves acceptably and reliably with changes in parameters and inputs, and whether the results are sensitive to changes in these variables. If any non-logical or inconsistent behavior is observed in the model, adjustments and refinements can be made to rectify these inconsistencies. Additionally, sensitivity analysis can help identify and manage risks associated with changes in parameters and inputs, leading to a better understanding of the system’s behavior.

##### Phase 3, Step 5 – model use, implementation, and dissemination

Once the model is tested and validated, it can be used to explore different scenarios and policy options related to the problem at hand. The model serves as a valuable tool for decision-making as it allows us to probe policies in advance and gain a better understanding of effective mechanisms and potential unexpected outcomes. The model functions as a virtual environment to test interventions and policies before real policy implementation.

##### Phase 3, Step 6 – design of learning strategy/infrastructure

Designing a learning strategy and infrastructure supports model use and dissemination. This includes creating training materials, user manuals, and policy briefs to facilitate the model’s application in policy circles. The model building process is iterative and flexible, allowing for continuous refinement based on feedback and new information. Ongoing improvements are possible as new data and understanding emerge.

#### Phase 4: presentation of policy interventions

The study will determine evidence-based policy interventions to address health workforce migration, prioritizing them using the Multi-Criteria Decision-Making method [36]. In this method, each option under consideration is evaluated based on different criteria determined by the decision makers. Critical criteria associated with the problem will be identified, including impact magnitude, sustainability, cost and benefit, social acceptance, international compatibility, feasibility, impact on income distribution, impact on economic growth, and compatibility with laws. Each option will be scored based on these criteria, and a prioritization matrix will be created to select the first option. Analytic Hierarchy Process (AHP) method helps us in making decisions. Decision maker compares the criteria and alternatives pairwise, assigning numerical values to their relative importance or preference using a scale. Through a series of calculations, the AHP determines the overall priority or weight of each alternative, helping to make more informed decisions [37]. Effective policies and solutions will be presented in a policy brief, including an introduction, problem definition, current situation analysis, policy objectives, strategies, budget, evaluation criteria, implementation measures, and recommendations for improved policy implementation. This approach allows decision-makers to compare options and identify the superior choice based on significant criteria [38].

## Discussion

Health worker migration, or ‘brain drain,’ poses a significant global health crisis and hinders global health equity. The medical brain drain has consequences on population health, training costs, and public health infrastructure in emigration countries. Resource-poor countries suffer from the loss of skilled health workers, leading to inadequate healthcare infrastructure and substantial financial losses [39]. Despite the negative impact on domestic health systems, mandatory national or return-of-service requirements may not deter migration. Gathering intelligence on how such requirements can incentivize health workers to stay is crucial [40]. Different analytical frameworks, such as the neoclassical approach, historical-structural approach, and migration systems theory, offer different policy responses to address health worker migration [41]. The effect of health worker migration on developing countries’ health systems is complex and multifaceted, involving factors such as individual rights, international mobility, and financial benefits from remittances [42]. Considering the high rate of intention to emigrate in the Iran’s health workers, a lot of whom will emigrate if their situation is ready, it can be a serious problem for Iran health system in the near future in which it will face lack of skilled health workers, and so requires more attention of health sector authorities [16]. In the present study, we will report on developing a whole-systems perspective of problems related to the emigration of health workforce in Iran in the future, what causes them, and how potential systems interventions can be identified and tested by our simulation model.

The research findings will be valuable for decision-makers in countries seeking to implement migration control policies. Governments and international agencies supporting these decision-makers can also benefit from the study’s outcomes. With over 30 countries facing health worker migration, these results have broad applicability [43–45]. The international community acknowledges the significance of studying migration dynamics, and system dynamics modeling provides a valuable approach to analyze the complex structure of migration systems. Simulations allow researchers to test hypotheses and understand the impact of modifying components, depicting migration as a system involving personal, social, and organizational elements [46,47].

While developing this approach, several limitations have been considered. Challenges include gaining access to healthcare professionals with experience in emigrating from Iran and obtaining their willingness to participate in interviews. Accessing records that provide insights into the scale of healthcare workforce migration in Iran is another limitation. Additionally, encouraging study participants to adopt a system thinking perspective can be difficult, especially for those accustomed to perceiving migration as a linear process. However, by using probes that promote holistic thinking and encourage participants to consider feedback relationships among components, researchers can foster a broader understanding of how migration is influenced by interconnected factors [48].

Finally, the results of this study have significant implications for Iranian policymakers, providing them with insights into the consequences of healthcare workforce emigration and offering guidance on effective interventions to prevent or minimize the emigration of Iranian healthcare workers. These findings can be instrumental in informing strategic planning efforts aimed at addressing the challenges associated with healthcare workforce migration in Iran.
